# Revealing the Hippocampal Connectome through Super-Resolution 1150-Direction Diffusion MRI

**DOI:** 10.1038/s41598-018-37905-9

**Published:** 2019-02-20

**Authors:** Jerome J. Maller, Thomas Welton, Matthew Middione, Fraser M. Callaghan, Jeffrey V. Rosenfeld, Stuart M. Grieve

**Affiliations:** 1 Sydney Translational Imaging Laboratory, Heart Research Institute, Charles Perkins Centre, University of Sydney, Sydney, Australia; 2 General Electric Healthcare, Richmond, Melbourne, Australia; 3 Monash Alfred Psychiatry Research Centre, Melbourne, Victoria, Australia; 4 Applied Science Laboratory, GE Healthcare, Menlo Park, CA USA; 5 Monash Institute of Medical Engineering, Monash University, Melbourne, Australia; 6 Department of Neurosurgery, Alfred Hospital, Melbourne, Australia; 7 Department of Surgery, F. Edward Hébert School of Medicine, Uniformed Services, University of The Health Sciences, Bethesda, MD USA; 8 Department of Radiology, Royal Prince Alfred Hospital, Sydney, Australia

## Abstract

The hippocampus is a key component of emotional and memory circuits and is broadly connected throughout the brain. We tracked the whole-brain connections of white matter fibres from the hippocampus using ultra-high angular resolution diffusion MRI in both a single 1150-direction dataset and a large normal cohort (n = 94; 391-directions). Using a connectomic approach, we identified six dominant pathways in terms of strength, length and anatomy, and characterised them by their age and gender variation. The strongest individual connection was to the ipsilateral thalamus. There was a strong age dependence of hippocampal connectivity to medial occipital regions. Overall, our results concur with preclinical and *ex-vivo* data, confirming that meaningful *in vivo* characterisation of hippocampal connections is possible in an individual. Our findings extend the collective knowledge of hippocampal anatomy, highlighting the importance of the spinal-limbic pathway and the striking lack of hippocampal connectivity with motor and sensory cortices.

## Introduction

The hippocampus is one of the oldest regions of the brain (the archicortex), and has wide-reaching connections. The hippocampus is considered the key component of the Papez circuit, which is generally described as the “emotional circuit” encompassing the medial temporal lobe (MTL: hippocampus, amygdala, and entorhinal cortex), hypothalamus, septum, prefrontal cortex, and the white matter (WM) circuitry connecting them. The hippocampus is implicated in a range of diseases and normal functions, however a detailed understanding of the structural connections of the hippocampus is currently lacking.

Most imaging studies of the hippocampus have focussed on either the morphology of the hippocampal formation or on intra-hippocampal microcircuitry. Macroscopic extra-hippocampal connections are less well described; likely due to technical limitations regarding how well these pathways can be evaluated. Compared to other major WM pathways of the brain (e.g. corpus callosum, cingulate bundle, and longitudinal fasciculi), the connections of the hippocampal formation to other brain regions are narrower and more tortuous^1^, therefore offering a greater challenge to techniques such as MRI. This has previously limited our ability to investigate the microstructure and connectivity of these pathways and, as a result, they are relatively poorly understood.

The impact of the hippocampus on various illness states may be as much a matter of “integrity” of connections as volume changes or morphometry. For example, Salat, *et al*.^2^ and Remy, *et al*.^3^ separately reported diffusional changes across the hippocampus and MTL, which were independent of grey matter (GM) degeneration or hippocampal volume. Whilst an atlas was recently released for the fornix, alveus and fimbria to facilitate volumetric investigations^4^ this does not illuminate hippocampal connectivity to other brain regions. Although there are a few studies illustrating hippocampal WM, these focus on intra-hippocampal structure, and required ultra-high magnetic field strengths (7 Tesla and above) and extremely high spatial resolution data (e.g. 100 µm isotropic voxels)^5,6^.

There are several investigations that have focussed on hippocampal connectivity to individual target regions with dMRI tractography techniques. For example, Dinkelacker, *et al*.^7^ investigated hippocampal-thalamic wiring in MTL epilepsy; Edlow, *et al*.^8^ studied tractography between the hippocampus and the human homeostatic network; Arrigo, *et al*.^9^ presented tractography data between the hippocampus, amygdala and brainstem (spinal-limbic pathway), and Rangaprakash, *et al*.^10^ tracked fibres connecting the striatum and hippocampal formation. There are also studies which specifically focus on connectivity between the hippocampus and the fornix^11^ and stria terminalis^12^. We are unaware of any published study that has conducted an unconstrained high angular resolution dMRI-tractography of the hippocampal formation (i.e. without endpoint *a priori* targets). As such, many other tracts connecting the hippocampus with the rest of the brain have not been investigated.

One recent study presented dMRI tractography of the human Papez circuit in considerable detail^13^, demonstrating connections among hubs in the MTL, retrosplenial gyrus, hippocampus, mammillary bodies, and anterior thalamic nuclei. The mammillary bodies are connected to the hippocampus via the fornix^1,14^, and directly to the anterior thalamic nuclei via the mammillothalamic tracts^15^. The Wei, *et al*.^13^ study acquired 514 diffusion directions at 1.5 mm isotropic spatial resolution. However, the tractography method used was deterministic (which does not consider crossing, abutting, or kissing fibres), tracked each pathway separately (as opposed to a single hippocampus seed), and generated only a limited number of streamlines (up to 10,000 per pathway).

Recent advances allow for the acquisition of diffusion MRI (dMRI) data using conventional human scanners with multiple diffusion strengths (“multi-shell”) and at multiple diffusion directions (and therefore greater “angular resolution”). In addition to improvements in dMRI acquisition, track-density imaging (TDI) and “super-resolution” analysis can provide greatly improved depictions of anatomical detail than standard maps based on eigenvalues^16^ to more clearly delineate WM fibres^17^. As shown in Supplemental Table 1, close to 30 published studies have used TDI to image the brain, although a third have been in non-human subjects. Together, these advances permit investigation of hippocampal connections in humans at a previously unseen level of detail.

Brain connectomics is a holistic approach to the study of brain connectivity in which pairwise measurements of connection strength are made between many brain regions. This allows the investigation of patterns of connectivity across the whole brain. This is especially pertinent given the recent shift in neuroscience toward a network-orientated view of brain structure and function. Major efforts have been given recently toward producing an accurate whole-brain connectome^18^, but such a study focussed on the hippocampus is lacking.

The aim of this study was to super-resolve *in vivo* human brain 3 T MRI diffusion tractography data from an ultra-high angular resolution dataset using track density imaging to compare it to *ex vivo* studies, with a focus on describing hippocampal connectivity. We then aimed to systematically evaluate the connections of the human hippocampus using a connectomic analysis of 166 regions of interest (ROIs) to study the strength and patterns of connectivity between the hippocampus and other brain regions.

## Results

### Global tractography

**Figure 1:**
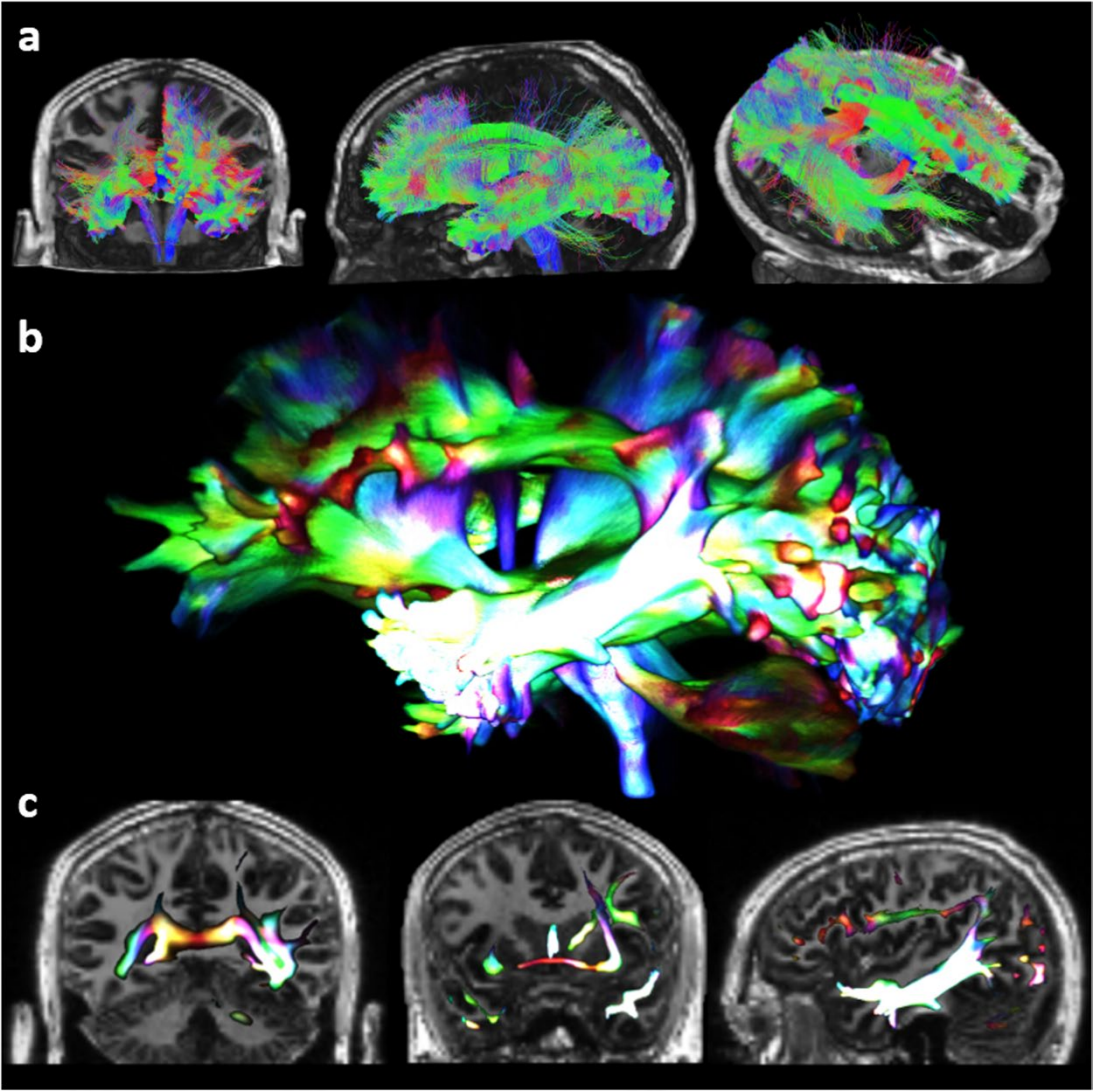
Tractography illustrating the major pathways of the hippocampal connectome. Whole brain tractography (**a**) used to create the track-density image (**b**). Selected slices of the track-density image (**c**), with directionally-encoded colour and brightness indicating track density, highlighting: tapetum (left), anterior commissure/mammillary bodies (middle), inferior longitudinal fasciculus (right).

Figure 1 shows the results of the unthresholded global tractography analysis of the left hippocampal connectome for the 1150-direction dataset. Data are displayed superimposed on a cut away T1-weighted image (Fig. 1a), as a 3D rendered track-density image (Fig. 1b), and as selected cross-sectional views of the track density image (Fig. 1c). These images demonstrate that the hippocampus is extensively connected both intra- and inter-hemispherically.

The ten most strongly connected ROIs accounted for 90% of total tracks. The large majority of regions were relatively weakly connected (156 of 166), with each having fewer than 1% of the total tracks. This suggests that the hippocampus is widely connected, but that the large majority of its connections are to a small number of regions.

Table 1 summarises the source-to-target connectivity for the 20 strongest connections (arranged by lobe). Tract numbers are quoted relative to the total number of hippocampal tracks reaching a GM target ROI, not the total number of seeded tracks. Approximately 70% of the total tracks reached a GM target ROI, with the remaining 30% terminating in WM, with the most common site of tracking failure being at the boundary of the lateral ventricles (75% of all maltracked fibres), largely secondary to partial volume effects decreasing the FA below our threshold.

**Table 1:** The 20 strongest connections of the left hippocampus, organised by lobe and ranked within each lobe by the track count.

Lobe	Target region	Strength (% tracks)	
		1150-direction dataset	CDCP
*Temporal*		**42.4**	**38.7 ± 8.0**
	Temporal pole	14.2	9.6
	Anterior transverse collateral sulcus	11.7	6.6
	Inferior temporal sulcus	0.4	1.1
	Inferior temporal gyrus	0.3	2.5
	Medial parahippocampal gyrus	15.8	18.9
*Limbic/Subcortical*		**33.8**	**29.1 ± 5.9**
	Mammillary bodies	3.6	0.7
	Amygdala	6.3	12.9
	Amygdala (contralateral)	0.3	0.1
	Thalamus	21.5	12.2
	Putamen	1.1	1.8
	Caudate	0.5	0.4
	Hippocampus (contralateral)	0.5	0.9
*Occipital*		**14.9**	**7.6 ± 4.3**
	Medial lingual sulcus	12.6	5.5
	Calcarine sulcus	0.8	1.1
	Medial lingual gyrus	0.8	0.4
	Occipital pole	0.4	0.3
	Occipital pole (contralateral)	0.3	0.3
*Frontal*		**3.1**	**1.6 ± 1.1**
	Subcallosal gyrus	1.8	0.8
	Pericallosal sulcus	1.3	0.8
*Parietal*		**0.7**	**0.4 ± 0.5**
	Parieto-occipital sulcus	0.7	0.4

All regions refer to the ipsilateral side except where stated. Bold rows show groupings of the connections by lobe, with the strength for these rows being the sum of the included connections with the sub-group.

**Figure 2:**
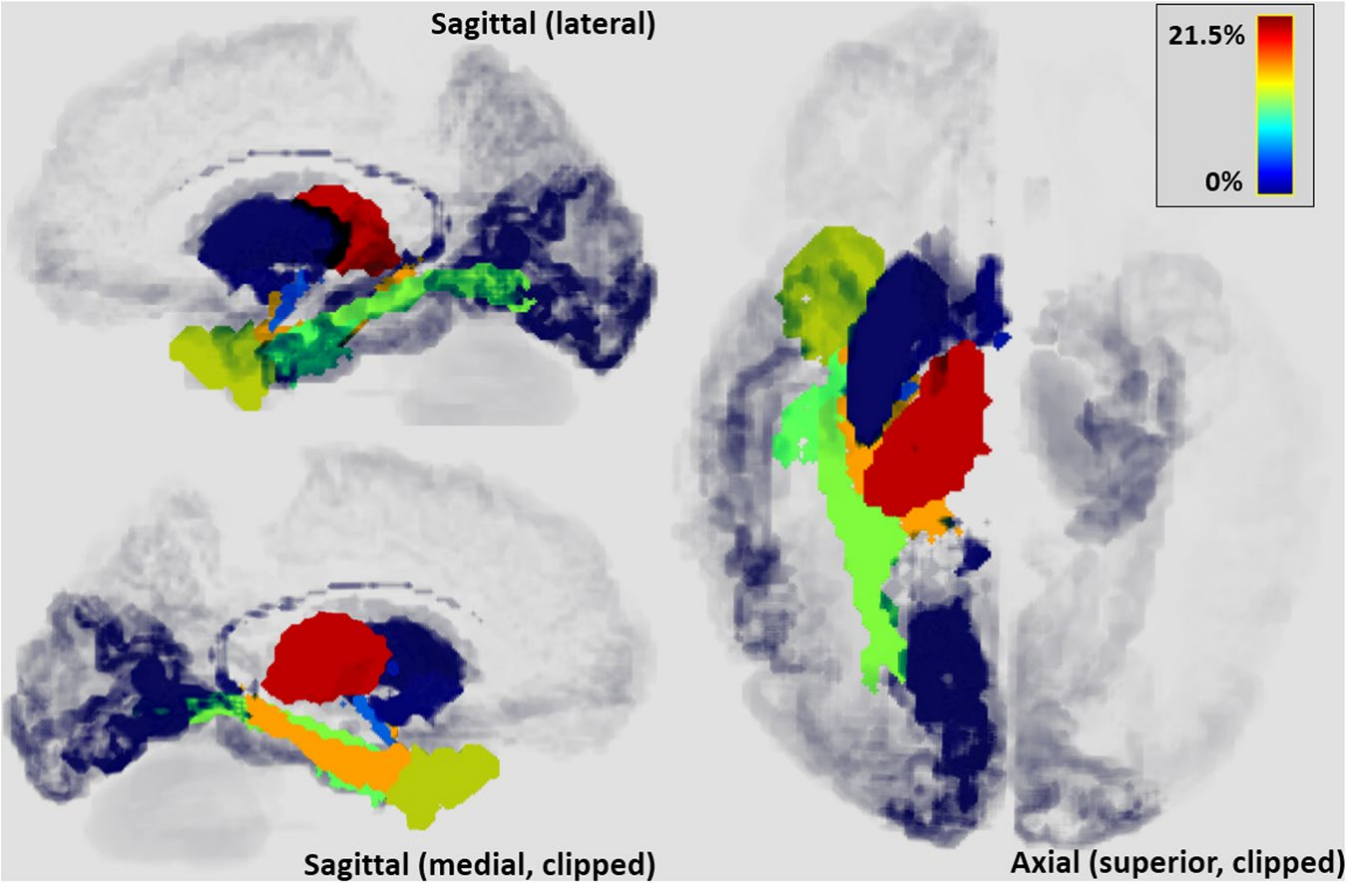
3D rendered ROIs with values equal to the normalised density of terminating tracks.

Figure 2 shows 3D renderings of the connection density, where the connection strength is colour-coded. The image reveals the high concentration of connections in temporal, limbic and sub-cortical regions, and the highly geographical nature of the distribution in the remaining cortex. By lobe, the strongest connection from the hippocampus was to the temporal lobe (42%), followed by the limbic/subcortical regions (34%), occipital lobe (15%) and relatively few connections to the frontal (3%) and parietal (1%) lobes. The strongest single connection in terms of number of tracks was between the hippocampus and the ipsilateral thalamus (21.5% of total tracks). The next strongest connections were to adjacent temporal structures (medial parahippocampal gyrus 15.8%, temporal pole 14.2%, anterior transverse collateral sulcus 11.7%), the medial lingual sulcus (12.6%) and to other limbic and subcortical regions (amygdalae 6.3% and 0.3%, mammillary bodies 3.6%, putamen 1.1%). Major tracts were also clearly demonstrated to the occipital pole (0.4%), putamen (1.1%) and caudate (0.5%) as well as to several other loci.

The connections were laterally symmetric, with clearly demonstrated ipsilateral and contralateral tracts extending from each hippocampus ROI. For clarity we therefore present only the left-sided data.

### Evaluation of Dominant Hippocampal Pathways

Inspection of the data revealed 6 long-range major hippocampal pathways which account for 96% of the total tracks. The six pathways are: (1) inferior longitudinal fasciculus (ILF), (2) spinal-limbic pathway (SLP), (3) anterior commissure, (4) cingulate bundle, (5) fornix and (6) tapetum. Table 2 summarises these pathways, with the top 20 strongest connections listed, grouped by pathway and ranked by strength (% of total intersecting tracks). For anatomical clarity and because of the numerical dominance of these pathways, we focussed our subsequent investigations on their relative strengths, anatomy, and regional connectivity.

**Table 2:** The 20 strongest connections of the left hippocampus, organised by pathway and ranked within each pathway by the median track length.

Pathway	Target region	Strength (% tracks)		Length (mm)	
		1150-direction dataset	CDCP	1150-direction dataset	CDCP
*Inf. longitudinal fasciculus*		**41.5**	**27.4 ± 6.9**		
	Temporal pole	14.2	9.6	68 (59–80)	68 (52–95)
	Medial lingual sulcus	12.6	5.5	61 (47–81)	54 (43–94)
	Anterior transverse collateral sulcus	11.7	6.6	64 (53–84)	66 (55–102)
	Calcarine sulcus	0.8	1.1	72 (63–105)	75 (62–122)
	Medial lingual gyrus	0.8	0.4	98 (76–115)	86 (67–125)
	Occipital pole	0.4	0.3	115 (96–123)	116 (86–133)
	Inferior temporal sulcus	0.4	1.1	70 (63–99)	70 (66–110)
	Inferior temporal gyrus	0.3	2.5	65 (56–105)	70 (58–103)
	Occipital pole (contralateral)	0.3	0.3	144 (130–170)	144 (115–159)
*Spinal-limbic pathway*		**23.1**	**14.4 ± 4.6**		
	Thalamus	21.5	12.2	56 (30–77)	59 (49–102)
	Putamen	1.1	1.8	106 (75–138)	107 (74–131)
	Caudate	0.5	0.4	122 (89–145)	119 (83–139)
*Anterior commissure*		**6.6**	**12.9 ± 4.2**		
	Amygdala	6.3	12.9	56 (48–65)	56 (45–70)
	Amygdala (contralateral)	0.3	0.1	173 (151–200)	159 (141–198)
*Cingulate bundle*		**3.8**	**2.1 ± 1.1**		
	Subcallosal gyrus	1.8	0.8	95 (80–129)	86 (63–115)
	Pericallosal sulcus	1.3	0.8	69 (29–118)	87 (62–145)
	Parieto-occipital sulcus	0.7	0.4	75 (67–107)	82 (70–130)
*Fornix*		3.6	**0.7 ± 0.5**		
	Mammillary bodies	3.6	0.7	101 (82–123)	108 (82–124)
*Tapetum*		0.5	**0.9 ± 0.9**		
	Hippocampus (contralateral)	0.5	0.9	105 (74–132)	119 (74–137)
*Short range*		15.8	**18.9 ± 5.8**		
	Medial parahippocampal gyrus	15.8	18.9	35 (15–54)	42 (30–71)

All regions refer to the ipsilateral side except where stated. Bold rows show groupings of the connections grouped by the dominant pathway in which they are included, with the strength for these rows being the sum of the included connections with the sub-group.

**Figure 3:**
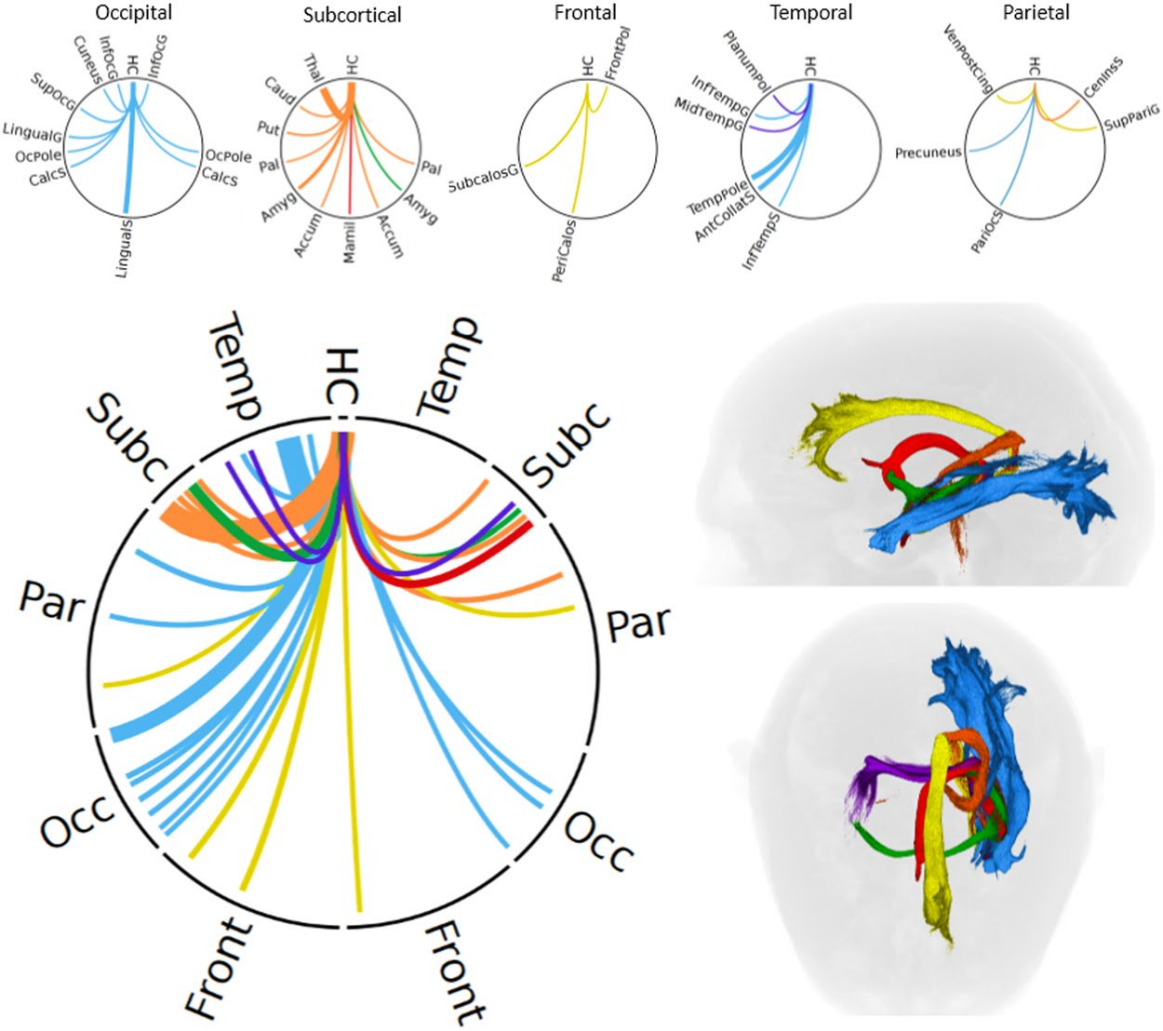
Summary of the hippocampal connectome (only left hippocampal connections shown for clarity). Ring plot showing connections having more than 0.1% of the total selected tracks, created using Circos (version 0.69^46^;). All ring plots are arranged symmetrically, with the left side representing the left brain hemisphere and vice-versa. Connections are grouped by colour according to the six observed major hippocampal tracts and correspond to the coloured tracts shown on the right 3D renderings (blue, inferior longitudinal fasciculus; orange, spinal-limbic pathway; yellow, cingulate bundle; green, anterior commissure; purple, tapetum; red, fornix). The five smaller ringplots (top) show focussed views of connectivity within each lobe. The spatial distribution of supra-threshold connections highlights the strong connectivity with bilateral subcortical, ipsilateral occipital and ipsilateral temporal regions, and relative weak connectivity with parietal and frontal regions (with exceptions). Abbreviations: HC, hippocampus; Temp, temporal; Subc, subcortical; Par, parietal; Occ, occipital; Front, frontal; Inf, inferior; G, gyrus; S, sulcus; Calc, calcarine; Sup, superior; Pal, pallidum; Amyg, amygdala; Accum, nucleus accumbens; Mamil, mammillary bodies; Put, putamen; Caud, caudate; Thal, thalamus; PeriCalos, peri-callosal; Subcalos, sub-callosal; Collat, collateral; Mid, middle; PlanumPol, planum polare; Cen, central; Ins, insular; Ven, ventral; Cing, cingulate.

Figure 3 shows a ring plot depiction of the hippocampal connectome, where connections are shown as curves between the source (12 o’clock position) and other regions organised radially around a circle. Data are grouped for clarity using the major hippocampal pathway involved (blue - inferior longitudinal fasciculus; orange - spinal-limbic pathway; yellow - cingulate bundle; green - anterior commissure; purple - tapetum; red - fornix).

The pathway with strongest connections was the ILF, which involved more than 40% of the total hippocampal tracks. ILF connections were primarily with the ipsilateral temporal and occipital lobes, and to the contralateral occipital lobe. Weaker connections involving the ILF were also present to the ipsilateral parietal cortex, including the parieto-occipital sulcus and angular gyrus.

The second most dominant pathway links the hippocampus with the thalamus, putamen, caudate and spinothalamic tract. We loosely use the term “spinal-limbic” pathway to describe this here due to the close correspondence to that described by Arrigo, *et al*.^9^. The SLP connections comprised approximately 23.1% of total connections, dominated by ipsilateral hippocampal-thalamic tracks (21.5%). Well-defined connections were also present to the putamen (1.1%) and caudate (0.5%).

The other pathways represent well-defined connections from the hippocampus to a single target ROI were demonstrated to the amygdala (6.0% and contralaterally via the anterior commissure, 0.3%), mammillary bodies (via the fornix, 3.6%) and to the contralateral hippocampus (via the tapetum, 0.5%).

The cingulate bundle carries relatively weak connections predominantly to the ipsilateral frontal (e.g. subcallosal gyrus, 1.8%; pericallosal sulcus, 1.3%), occipital and parietal cortex (parieto-occipital sulcus, 0.7%; superior parietal gyrus, 0.1%). We found a negligible number of tracks to pre- or post-central gyri (<0.005%), which could be considered noise. A large number of “short range” connections were also measured to the parahippocampus (15.8%), which we classify separately.

Distributions of track lengths are shown in Supplemental Fig. 1. Over 80% of tracks are greater than 2 cm in length, and a considerable number of long range tracts were measured (>25 cm; 5%). The major tracts described above correspond to clearly defined peaks within these histograms.

In keeping with our findings in the lobar analysis, we found evidence of age and gender effects across these pathways. These findings are elaborated under each pathway heading.

### Generalisation and Creation of a Reference Connectome

In the CDCP cohort, the average age was 32 years (standard deviation 16) and 54% of the cohort was female. The overall pattern of connectivity for the CDCP group was concordant with the 1150-direction dataset. The distributions matched well in terms of their rank, with the top 10 strongest regions being the same for both 1150-direction dataset and CDCP. Generally, however, the connection strength distribution in the CDCP was somewhat “flattened”, with the strongest connections being weaker (e.g. to the temporal pole with a difference of 4.6%) and the weaker connections being slightly stronger (e.g. to the contralateral hippocampus with a difference of 0.5%). Variation in connection strengths in the CDCP was generally high, e.g. the coefficient of variation ranged from ~30% to ~100%. Histograms of connection strength across the cohort have log-normal distributions.

Among the CDCP cohort, we found significant associations between hippocampus-parietal lobe connection strength and age (F = 6.95; p < 0.001; significant after controlling for the false discovery rate; with older subjects having stronger connectivity) and between hippocampus-temporal lobe connection strength and gender (F = 4.41; p = 0.041; non-significant after controlling for the false discovery rate; with females having stronger connectivity; Supplemental Fig. 2).

To facilitate use of the high resolution single subject and anatomical population data, we created an open-resource CDCP Hippocampal Connectome (CDCP-HC) in the form of downloadable 3D NIFTI volumes and associated CSV files (https://www.stil.net.au/downloads). The CDCP-HC Pathway volume reflects track density for each of the 6 pathways and the ROI volume includes segmented FreeSurfer ROIs with added mammillary bodies and brainstem masks in MNI space, with each ROI having a value equal to the proportion of intersecting tracks from the hippocampus. The first volume is the mean track density and the second volume is the standard deviation. The CSV file provides the same data, and has a 2 × 166 shape, with the dimensions representing mean and standard deviation for each of the 166 included ROIs, respectively.

### Detailed evaluation of the Hippocampal Connectome by Pathway

#### Inferior Longitudinal Fasciculus

**Figure 4:**
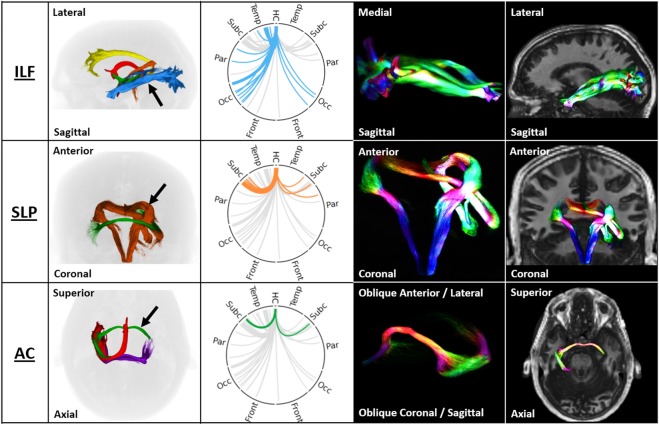
Coupled ring plot connectome and track density depictions of the inferior longitudinal fasciculus (ILF), spinal-limbic pathway (SLP) and anterior commissure (AC). The first column shows three views of these pathways, revealing the relative orientation and anatomy. Pathways are color-coded consistent with the connectome ring plot (orange = SLP; blue = ILF; green = AC). The 2nd column shows ring plot connectomes for each pathway following this colour convention. The 3rd and 4th columns show 3D depictions of the geometry of each isolated tract (3rd column), and then superimposed on a rendered image of the brain to illustrate the relative location of the pathway (4th column).

The ILF (as shown in Fig. 4) consists of short and long association fibres originating in the middle and inferior temporal and parahippocampal gyrus, hippocampus, amygdala, and temporal pole and terminate in the extrastriate areas, consistent with previous descriptions^19,20^. The supra-threshold regions connected to the hippocampus which involved the ILF were predominantly ipsilateral temporal and occipital regions, with relatively weak connections to ipsilateral parietal and contralateral occipital regions (Table 2). The median track length was 133 mm and the distribution was highly leptokurtic, with a tail longer on the left than the right. An interesting feature of the tract shape is its splitting into three smaller intertwined bundles, which has not previously been visualised. The most superior of these may constitute fibres which follow the “temporal loop” of the optic radiation^20^. The ILF was the least variable of the pathways, with a coefficient of variation of 25%. Within the ILF, strength of connectivity to the calcarine sulcus, inferior temporal gyrus, medial lingual gyrus and occipital pole target regions was strongly positively correlated with age (Table 3). Connectivity to the inferior temporal gyrus was significantly stronger in females (t = −3.14, p = 0.003; Table 3).

**Table 3:** Tests for age and gender effects calculated using the means of the included connections. Correlations between age and connection strength in the CDCP cohort are presented as the Pearson correlation coefficient (r) and uncorrected p-value.

Pathway	Target Region	Age (r, uncorrected p)	Gender (t, uncorrected p)
*Inf. longitudinal fasciculus*		**−0.21, 0.118**	**−1.60, 0.117**
	Temporal pole	**−**0.10, 0.438	**−**0.11, 0.913
	Medial lingual sulcus	**−**0.09, 0.497	**−**0.27, 0.784
	Anterior transverse collateral sulcus	**−**0.16, 0.230	**−**1.76, 0.086
	Calcarine sulcus	0.39, 0.004**	1.60, 0.116
	Medial lingual gyrus	0.44, 0.001**	1.03, 0.306
	Occipital pole	0.32, 0.018**	2.26, 0.030*
	Inferior temporal sulcus	**−**0.27, 0.043*	**−**0.80, 0.425
	Inferior temporal gyrus	**−**0.34, 0.011**	**−**3.14, 0.003**
	Occipital pole (contralateral)	**−**0.12, 0.387	**−**0.95, 0.347
*Spinal-limbic pathway*		**−0.16, 0.239**	**0.70, 0.484**
	Thalamus	**−**0.20, 0.143	0.28, 0.777
	Putamen	0.03, 0.794	1.25, 0.215
	Caudate	0.09, 0.488	2.27, 0.028*
*Anterior commissure*		**0.25, 0.071**	**0.96, 0.339**
	Amygdala	0.25, 0.071	1.00, 0.320
	Amygdala (contralateral)	**−**0.01, 0.930	**−**1.26, 0.216
*Cingulate bundle*		0.38, 0.005**	**0.95, 0.346**
	Subcallosal gyrus	0.11, 0.405	0.78, 0.439
	Pericallosal sulcus	0.22, 0.103	**−**0.27, 0.784
	Parieto-occipital sulcus	0.31, 0.020**	1.96, 0.057
*Fornix*		**−0.20, 0.151**	**0.16, 0.866**
	Mammillary bodies	**−**0.20, 0.151	0.16, 0.866
*Tapetum*		**−0.12, 0.393**	**−1.02, 0.311**
	Hippocampus (contralateral)	**−**0.12, 0.393	**−**1.02, 0.311
*Short range*		**−0.00, 0.989**	**−0.61, 0.544**
	Medial parahippocampal gyrus	**−**0.00, 0.989	**−**0.61, 0.544

*p < 0.05 (uncorrected); **significant after controlling for a false discovery rate of 0.1.

Gender effects were evaluated using a two-tailed independent-samples t-test, with the t-statistic and uncorrected p-value reported in the table. Statistical tests were controlled for multiple comparisons using the false discovery rate method with an expected false discovery rate of 0.1.

#### Spinal-Limbic Pathway

Of the six identified bundles, the SLP has the most complex shape. The SLP extends from the brain stem to the amygdala and along the hippocampal body, consistent with Arrigo, *et al*.^9^. It then merges with the tapetum and crosses into the contralateral hemisphere, forming an “ivy leaf” shape in the coronal view, as shown in Fig. 4. This pathway connects brainstem, hippocampus and subcortical nuclei: thalamus, putamen and caudate (Table 2). While the hippocampus-thalamus connection via the SLP is very strong, the SLP is otherwise the least-dense of the six major bundles, which is a likely contributor to the relative lack of literature surrounding this pathway. The track length distribution was normal, with a single peak at 167 mm and long tails. In the CDCP cohort, the strength of the SLP had a relatively low coefficient of variation (32%). None of the involved connections were significantly associated with age or gender, except the caudate nucleus with gender, but this did not survive after correction for multiple comparisons.

#### Anterior Commissure

The anterior commissure was revealed as a narrow yet dense pathway between the anterior hippocampi and amygdalae as illustrated in Fig. 4. At each end of the commissure, fibres spread into a fan-like shape. Track lengths ranged from 100 mm to 350 mm, with multiple distinct peaks within that range. The anterior commissure had a relatively low variation in connection strength, with a coefficient of variation of 32%, and was not associated with either age or gender differences.

#### Cingulate Bundle

**Figure 5:**
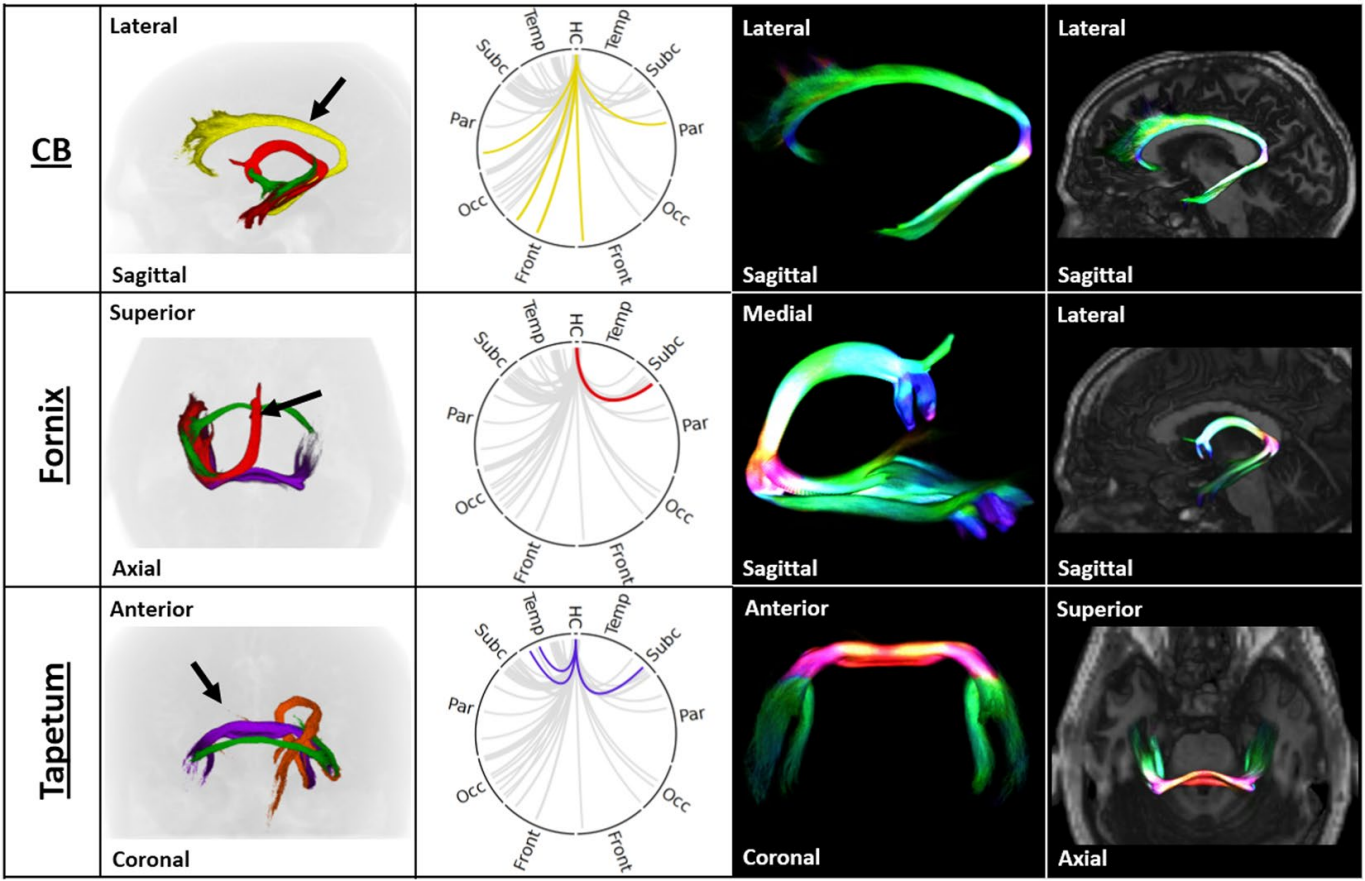
Track density images of the cingulate bundle (CB), fornix and tapetum. The first column shows three views of these tracts, revealing the relative orientation and anatomy. Tracts are color-coded consistent with the connectome ring plot (yellow = CB; red = fornix; purple = tapetum). The two right columns show 3D depictions of the geometry of each isolated tract (third column), and this tract superimposed on a rendered image of the brain to illustrate the relative location of the tract (far right column).

The cingulate bundle is depicted in Fig. 5, and extends from the anterior hippocampus to the subgenual region in parallel to the long body. Tracks from the hippocampus which traversed the cingulate bundle primarily reached the medial frontal, parietal and subgenual cortex with near zero branching into motor and sensory cortices. Tracks had an average length of 170 mm. The cingulate bundle was slightly more variable than the above pathways in hippocampal connection strength (coefficient of variation 52%) and, in combination, was significantly positively associated with age (r = 0.38, p < 0.005; Table 3). This relationship was mostly driven by the connection to the parieto-occipital sulcus.

#### Fornix

The contiguous alveus (head), fimbria (midbody), and fornix (posterior) form a major WM bundle, which merges into a laterally orientated arch shape to become the fornix medially (Fig. 5). At its rostro-dorsal tip (colored blue in Fig. 5), the fornix terminates in the mammillary bodies. The fornix is the shortest of the six bundles, with the largest peak being at 90 mm and a smaller peak at 130 mm. Perhaps due to its narrow, curved shape, the fornix was more variable than most other pathways (coefficient of variation, 71%). We found no relationship between fornix strength and either age or gender.

#### Tapetum

The tapetum borders the splenium of the corpus callosum and radiates in a fan-like shape (Fig. 5). The tapetum connects the hippocampi bilaterally. Tracks at either end of the tapetum formed two distinct branches, shown as two peaks in the histogram of track lengths at ~80 mm and ~120 mm. As the basic diffusion data (FODs) representing the tapetum and corpus callosum co-exist within single voxels, the probabilistic tractography technique employed therefore propagated along both of their bundles. Essentially, the tapetum appears as a continuation of the corpus callosum. The failure to resolve these structures even at a super-resolution of 300 µm demonstrates the extremely close adjacent positions of these structures. The tapetum was the most variable of the six pathways in the CDCP cohort (coefficient of variation, 100%), although this may also be due to the difficulty of imaging this structure.

## Discussion

We present the most comprehensive, highest angular resolution map of macroscopic hippocampal connections to date. Our data demonstrate that accurate *in vivo* diffusion MRI tractography of the hippocampus is possible with conventional MRI hardware. The results from the 1150-direction dataset are clearly meaningful at an individual level, providing concordant measurements with both our large normal cohort and against previous human and *ex-vivo* preclinical studies. Our results demonstrate the benefits of high angular resolution sampling of diffusion data in both angularity and diffusion weighting, and illustrate that large scale measurements of cohorts is possible, and likely necessary for further extending our detailed understanding of pathobiology involving the hippocampus. Finally, our data reveal three key anatomical findings: (1) the hippocampal connectome is more extensive than has previously been demonstrated *in vivo*, (2) one of the major hippocampal connections is a spinal-limbic pathway, which has not previously been studied, and (3) in contrast to previous functional connectivity studies, there was almost no connectivity with motor or sensory cortices.

Our analysis revealed a wide network of hippocampal connections. We identified several relatively strong connections, which had previously not been described in detail using *in vivo* data. For example, the connectivity between the hippocampus and ILF has rarely been touched upon in the literature, and the spinal-limbic pathway has only recently been reported upon using *in vivo* data by Arrigo, *et al*.^9^. While the hippocampal network is extensive, it is centred on few strongly-connected limbic and subcortical structures which likely serve as hubs for a variety of functions. These hubs are mostly centred on the Papez circuit. We ranked connections to individual brain regions by strength and, from this, could isolate six distinct long-range pathways representing the majority of total hippocampal connections: ILF, SLP, anterior commissure, cingulate bundle, fornix and tapetum. We further characterised each of these in detail using qualitative analysis of super-resolved track density images and quantitative information on connectivity strength and tract length.

Our connectome measurements in the 1150-direction dataset were mirrored in the CDCP cohort of 94 datasets and corresponded to evidence from preclinical and *ex-vivo* studies using neuronal tracers. Our CDCP Connectome data showed considerable variation in fibre track strength in some connections, and highlight the influence of gender and age. These latter findings require further detailed study and correlation with measures of functional performance or disease risk. Our optimised protocol is 391-directions, exceeding any major database yet acquired. The intra-cohort correlation and the correspondence with our ultra-highly sampled 1150-direction dataset suggests that such a single subject high angular resolution dataset is likely accurate enough to give a meaningful indication of tract strength and course across the brain. Our prior analysis demonstrates that, at lower angular resolutions, longer and more tortuous pathways are poorly-tracked^21^. This speaks to an increased emphasis on better phenotyping and the possibility of much smaller required sample sizes, and suggests that, with further improvements to MRI acquisition and analysis techniques, imaging might feasibly contribute to a personalised clinical approach in future.

The variation in the CDCP cohort is likely due to both the natural population variation in connection strength, and to the expected poorer tracking performance in the 391 CDCP data^21^. In particular, we expect that the false negative rate was higher, with more tracks which would usually go directly to their “true” target, terminating without reaching it, leading to the flattened distribution around a less strong peak. An additional separate issue is that narrow bundles, e.g. fornix and anterior commissure, tracked more poorly in the CDCP cohort, a systematic difference which would not be solved by averaging over the large CDCP sample. Aside from the angular resolution difference, a likely contributor to this effect is a probable higher level of motion in the CDCP cohort compared to the 1150-direction acquisition.

By releasing our connectome results as an open downloadable resource, we hope that others might expand on this work and that our parcellation might serve as a reliable precedent. We aim to update the CDCP-HC dataset when more data become available from this project.

**Figure 6:**
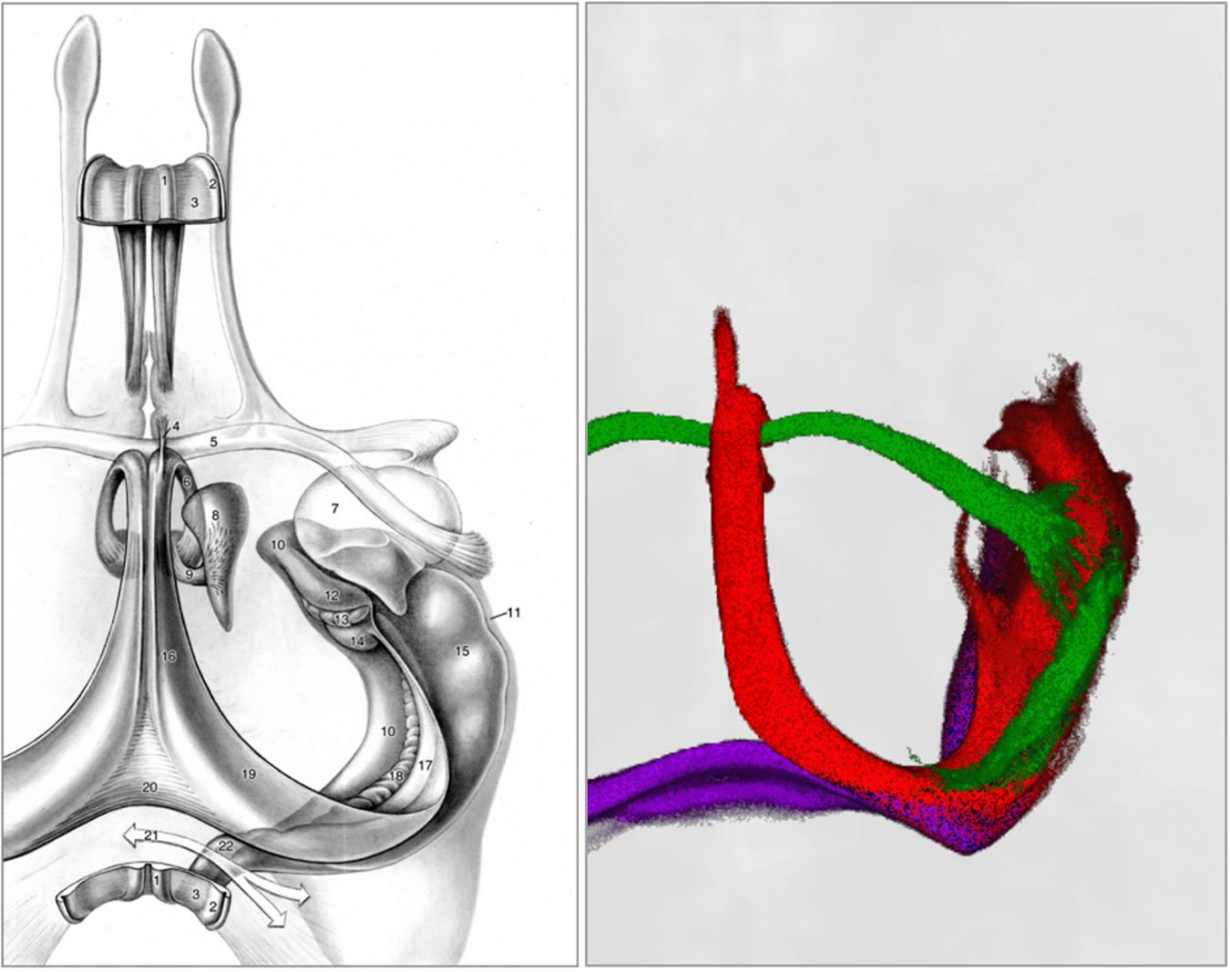
Comparison between diffusion MRI tractography analysis (right) and an illustrated dissection of the hippocampal formation and other limbic structures isolated from most of their surroundings, seen from above. Drawing (left) reproduced from Nieuwenhuys, *et al*.^1^, with permission. Labels: 1, medial longitudinal stria; 2, lateral longitudinal stria; 3, indusium griseum; 4, precommissural fornix; 5, anterior commissure; 6, column of fornix; 7, amygdaloid body; 8, anterior thalamic nucleus; 9, mamillothalamic tract; 10, subiculum; 11, lateral ventricle, inferior horn; 12, Ammon’s horn (uncinate gyrus); 13, limbus giacomini; 14, Ammon’s horn (intralimbic gyrus); 15, ammon’s horn (hippocampal digitations); 16, body of fornix; 17, fimbria of hippocampus; 18, dentate gyrus; 19, crus of fornix; 20, commissure of fornix; 21, site of corpus callosum; 22, fasciolar gyrus. Colours: red, fornix; green, anterior commissure; purple, tapetum.

Consistent with previous *in vivo* studies, the hippocampus was shown to be connected to the parahippocampal gyrus, fornix, thalamus and mammillary bodies, as illustrated in Fig. 6. The cingulate bundle is often considered part of the fronto-temporal/limbic network and the Papez circuit, as was confirmed in our hippocampus super-resolution images. A recent study on Papez circuit tractography^13^ failed to demonstrate a direct connection. Indeed, the hippocampus is not directly connected to the cingulate (the cingulate gyrus borders the vestigial precommissural and supracollosal parts of the hippocampus^1^), but transmits to it via the PHG. Similarly, Granziera, *et al*.^22^ tracked WM bundles involved in the Papez circuit but did not find the cingulate connected directly to the hippocampus. We recently performed tractography of the cingulum bundle in the 1150-direction dataset presented here^21^, and were able to demonstrate connectivity between them, probably due to higher angular and diffusion sampling resolution.

Whilst regions other than those above have been shown in previous tractography studies, this is the first to show them all from a single dataset demonstrating the extensive hippocampal network. Apart from the PHG, fornix, and mammillary bodies, streamlines extended to the tapetum, septum pallucidum, anterior commissure, external capsule, posterior limb of the internal capsule, superior longitudinal fasciculus, inferior longitudinal fasciculus, optic radiation, lateral olfactory stria, and lateral geniculate nucleus. These are structures beyond those described in the existing literature in the context of the conventional hippocampal, or limbic network. Furthermore, some of them are considered among the most difficult to observe with tractography, such as the anterior commissure^23^. We were also able to reveal the so-called “spinal-limbic” pathway, which has received relatively little attention. Arrigo, *et al*.^9^ recently demonstrated this circuit for the first time in a human using a 3 T MRI scanner, 64 diffusion directions and a single-shell acquisition scheme (b = 1500), revealing fibres extending from the amygdala and hippocampus to the cervical spinal cord. It is known that brainstem nuclei are linked to the hippocampus^24,25^ and, by using a multi-shell acquisition with higher b-values, we were able to show its full extent of connectivity. In small animals, it is reportedly to be involved in pain transmission^26^ and that visceral activity may influence higher neurobehavioral processes^27^.

The hippocampus precommissuralis (situated at the ventral-anterior end of the cingulum) was not revealed in the tractography, likely because it is a thin WM strand beyond the spatial resolution of our data (extending throughout the length of the corpus callosum^1^;). However, it is a part of the accessory optic system involved in the optic flow to the hippocampal formation^28^, and this is likely to have contributed to the tracking on the optic pathways in this study.

We found significant associations to age and gender in several hippocampal pathways. Older age was associated with stronger connectivity primarily to medial occipital regions including the parieto-occipital sulcus, calcarine sulcus, medial lingual gyrus and occipital pole. These findings agree with previous reports that long-range ipsilateral connectivity tends to increase with age, while inter-hemispheric connectivity decreases^29,30^. Connectivity to the inferior temporal gyrus was significantly stronger in females, which is also consistent with functional and structural studies of hippocampal connectivity^29^. Previous studies of these specific pathways only investigated tract fractional anisotropy^31,32^ and may not be reliable due to methodological limitations. Further, these relationships are likely non-linear^32,33^. The drivers for these effects are not fully understood but are thought to be related to over-connectivity followed by pruning in neurodevelopment^34^. This phenomenon has been observed at a network level in terms of development from a more locally-connected pattern to a more widely-distributed pattern of connectivity^35^.

For tractography, the minimum number of diffusion directions required for a robust estimation of tensor-orientation and mean diffusivity has been suggested to be 30 directions^36^, whilst Tournier, *et al*.^37^ reported the minimum number of directions required to adequately model the diffusion signal to be 45. As we previously described^21^, the number of directions has a direct impact upon tractography as more directions yields greater angular resolution, although it is always a compromise between spatial and angular resolution as increasing either results in longer scan time and potentially increases motion effects. We previously found substantial (up to 34-fold) improvements in tracking accuracy using a dataset comprised of 1150 diffusion directions when compared to a 64-direction dataset, while 140-direction data offered a maximum 17-fold improvement. Our study showed that high angular resolution is required to elucidate fine connections between regions and that lower angular resolution data may introduce systematic anatomical biases. It may, therefore, be pertinent to investigate the trade-offs among angular and spatial resolution, signal-to-noise and distortion to identify whether there is a sweet spot for diffusion image acquisition parameters.

Future studies should include a greater number of subjects to consider validity and reproducibility, and the inclusion of clinical subjects will create the opportunity to investigate the effect of diffusion directions upon accuracy of probing diagnosis, treatment, and outcome. As MRI technology continues to develop, the capability to acquire ultra-high angular and spatial resolution dMRI datasets in clinically feasible times will become more and more accessible, allowing for remarkably detailed information of connectivity on an individual level. Ultra-high magnetic field is also expected to confer significant improvements in spatial resolution. The combination of super-resolution diffusion MRI with functional MRI, PET and depth electrode hippocampal EEG recordings could improve diagnostic capability and precision in neurosurgical applications, such as temporal lobe epilepsy and tumour surgery and radiosurgery.

Hippocampus subfield connectivity to conventionally examined limbic and subcortical structures has been described and eloquently detailed in a recent review^38^, albeit mostly based on fibre dissection techniques. There was not sufficient signal in our dMRI data to examine hippocampus subfield connections to other regions, even at a “super resolution” of 300 µm. However, as demonstrated with the cingulum bundles, we may be able to produce streamlines from the subfields but they would likely be too sparse or of too low density to be revealed, as the super resolution technique is based on density. Furthermore, Beaujoin, *et al*.^5^ demonstrated that the intra-hippocampal connectivity is highly complex in that most subfields are invariably connected to one another. Hence, tractography of any one specific subfield would likely be similar to tractography of any other specific subfield in terms of connectivity to extra-hippocampal regions. Acquiring dMRI data with multiple averages will enhance the signal, as would utilising a stronger magnet (e.g. 7 Tesla), and this is an aim of our future investigations.

This study found that detailed visualisation of WM connectivity to extra-hippocampal regions is possible with ultra-high angular resolution diffusion data super-resolved to 300 µm. Our findings substantially build upon previous reports of these networks and are consistent with and extend prior MRI and post-mortem *ex vivo* studies. It therefore creates a benchmark for future *in vivo* MRI research efforts into elucidating WM trajectories and connectivity with brain regions through mapping of the hippocampal connectome.

## Methods

### Subjects

One healthy 42 year-old male subject underwent a single-session MRI assessment at Macquarie Medical Imaging, Sydney, New South Wales. A further 94 healthy subjects from the Chronic Diseases Connectome Project (CDCP) underwent a standardised exam including a 391-direction dMRI dataset. Subjects had no history of psychiatric, neurological or cardiac disorder, and no contraindication to MRI scanning. Subjects gave informed consent and the study had ethical approval from the Adventist HealthCare Limited Human Research Ethics Committee and the Macquarie University Medical Sciences Human Research Ethics Committee. All experiments were performed in accordance with the relevant guidelines and regulations.

### Image Acquisition

The 1150-direction diffusion MRI sequence has been described in detail previously, and was shown to confer between 11 and 34 fold improvements in tracking accuracy over a conventional 64-direction scan, dependent on tract anatomy^21^. Data were acquired using 3 Tesla GE MR750w Discovery MRI scanner (GE Healthcare, Milwaukee, Wisconsin) with DV25.1 software and a 32-channel Nova head coil. A HyperBand blipped-CAIPI sequence was employed with 1150 unique diffusion directions over 11 shells (maximum b-value = 2800) and 65 b = 0 volumes interspersed throughout the acquisition using a 2 mm isotropic voxel size. An additional b = 0 volume was acquired with the phase encoding polarity reversed. The participants from the CDCP cohort underwent a similar highly-optimised clinically-practical diffusion sequence using the same parameters but with 391 unique diffusion directions over 11 shells with a maximum b-value of 2800 and 22 b = 0 volumes. The acquisition time was 25 minutes.

In all subjects, we also acquired a contiguous AC-PC aligned sagittal MPRAGE PROMO (PROspective MOtion correction) T1-weighted volume (TR = 10.172 ms, TE = 4.064 ms, TI = 500 ms, flip angle = 8 degrees, matrix = 284 × 284, field of view = 256 mm, voxel dimensions = 0.9 mm isotropic). Total scanning time was 90 minutes for the 1150 individual brain, and 35 min for each CDCP subject.

### Image Analysis

The T1-weighted data were processed in FreeSurfer (version 6^39^;) to segment the brain into 166 cortical and subcortical regions of interest (ROIs) based on the atlas of Destrieux, *et al*.^40^. The FreeSurfer brainstem segmentation was run in order to generate a custom pontine ROI. Because FreeSurfer does not produce a mask of the mammillary bodies, this was generated by manually drawing a mask using the hippocampus TDI as a guide and added to the other FreeSurfer masks.

For the hippocampi, we combined hippocampal subfield ROIs, excluding the hippocampal fissure^41,42^. A rigid-body transformation was created using the T1-weighted image and the first b = 0 volume, which was then applied to the ROIs to register them into diffusion space.

Diffusion data were pre-processed as described previously^21^. The FMRIB’s Automated Segmentation Tool (FAST^43^;) was then used to create whole-brain WM, GM and CSF masks. These were used to create response files for each tissue type using appropriate harmonic order numbers based on the parameters of each combination of diffusion directions. Based on the response files, fibre orientation distributions (FODs) were created using the MRtrix3 constrained spherical deconvolution iFOD2 technique^44^. Fibre tracking was performed, seeding 50 million unconstrained tracks bidirectionally from each hippocampus ROI. Tracking was terminated when the angle between successive steps exceeded 45° or the track exited the brain volume. Tracks shorter than 10 mm were excluded.

We generated three main outputs:

1. The hippocampal connectome was first defined using the 1150-direction dataset. Tracks were generated to every GM ROI. We then ranked each hippocampus-to-ROI connection by the number of intersecting tracks to that ROI out of the number of total successful tracks (all those which reached a GM ROI). Based on these data, six “dominant” pathways were defined to categorise the 20 strongest connections. To measure the track counts in these six classical (“dominant”) pathways, we first classified each of the 20 strongest hippocampus-to-target tracts into one of the six groups. This was performed with the neuroanatomical and neuroradiological expertise of authors JJM and SMG. Then, the sum of the track counts in the component tracts of each group was calculated to give a total track count for each dominant pathway.
2. Our single-subject connectome analysis was then tested for generalisability in the CDCP cohort. This included an exploratory analysis of age and gender effects in relation to the strength of connections to each lobe using a general linear model. Tract-wise connection strength was quantified by the track count between a pair of regions averaged across the cohort. Lobe-wise connection strength was quantified by taking the sum of tracks across all tracts innervating the lobe and averaging these across individuals. We also created an open resource reference CDCP Hippocampal Connectome including downloadable images and matrices (CDCP-HC; https://www.stil.net.au/downloads).
3. Anatomical evaluation of these hippocampal connections was then performed using super-resolved track-density images at 300 µm. Super-resolution track density imaging uses the sub-voxel information available from the fibre tracks to generate images of a higher resolution than the acquired diffusion MRI data. To create these, the tractogram was converted into a map of the fraction of tracks to enter each voxel^45^. These were used for qualitative assessment of hippocampal anatomy by experts, JJM, SMG and JVR.

## Supplementary information

Supplemental Information

## Data Availability

The datasets generated during and/or analysed during the current study are available from the corresponding author on reasonable request.
