# Supplementary material for: Revealing the Hippocampal Connectome through Super-Resolution 1150-Direction Diffusion MRI
Source: Sci Rep. 2019 Feb 20;9:2418. doi: 10.1038/s41598-018-37905-9 (PMC6382767; doi:10.1038/s41598-018-37905-9)
Supplement: Supplementary file 1 — Supplemental Information [file 41598_2018_37905_MOESM1_ESM.docx]

**Supplemental Information**

Revealing the Hippocampal Connectome through Super-Resolution 1150-Direction Diffusion MRI

Jerome J. Maller^§,1,2,3^, Thomas Welton^§,1^, Matthew Middione^4^, Fraser M. Callaghan^1^, Jeffrey V. Rosenfeld^5,6,7^, Stuart M. Grieve^1,8,*^

^1^ Sydney Translational Imaging Laboratory, Heart Research Institute, Charles Perkins Centre, University of Sydney, Australia.

^2^ General Electric Healthcare, Richmond, Melbourne, Australia.

^3^ Monash Alfred Psychiatry research centre, Melbourne, Victoria, Australia.

^4^ Applied Science Laboratory, GE Healthcare, Menlo Park, CA, USA.

^5^ Monash Institute of Medical Engineering, Monash University, Melbourne, Australia.

^6^ Department of Neurosurgery, Alfred Hospital, Melbourne, Australia.

^7^ Department of Surgery, F. Edward Hébert School of Medicine, Uniformed Services, University of The Health Sciences, Bethesda, MD, USA.

^8^ Department of Radiology, Royal Prince Alfred Hospital, Sydney, Australia.

^§^ Co-first authors.

^*^ Corresponding author.


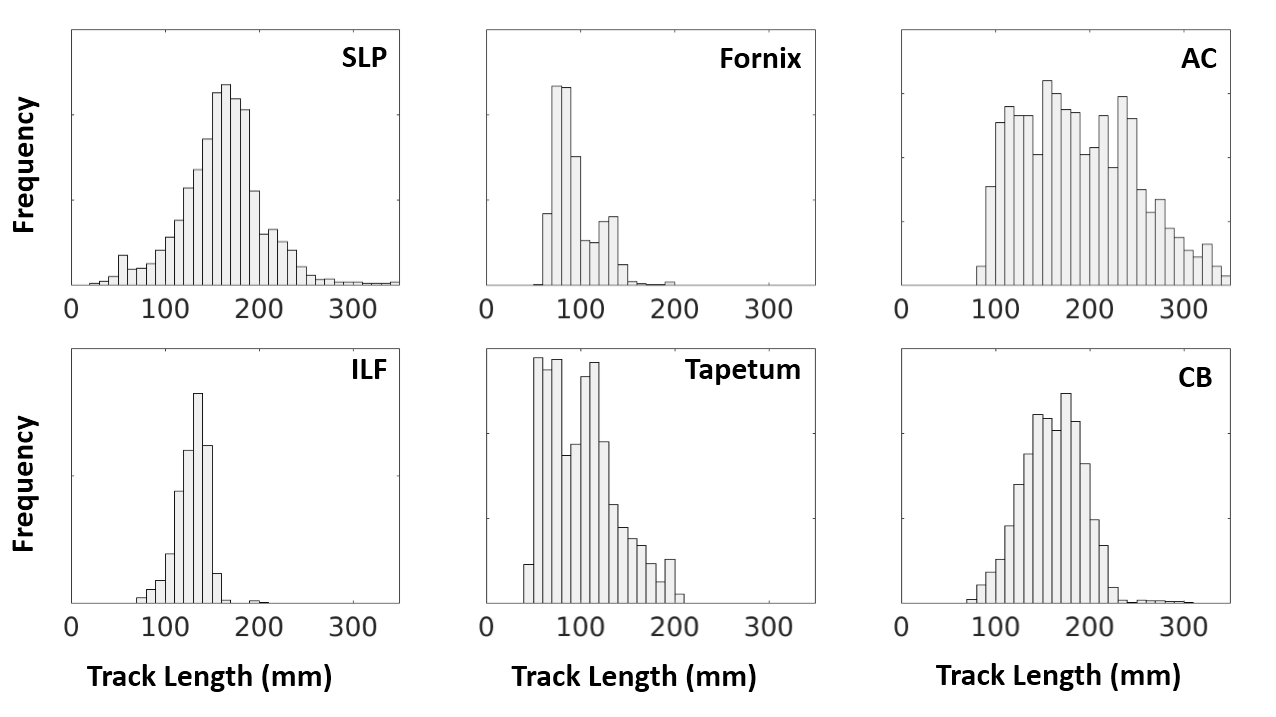


*Supplemental Figure 1.* *Histograms of track lengths in each of the six major hippocampal pathways. Data from the 1150-direction dataset.*


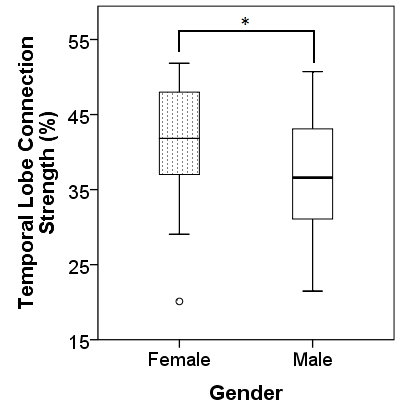

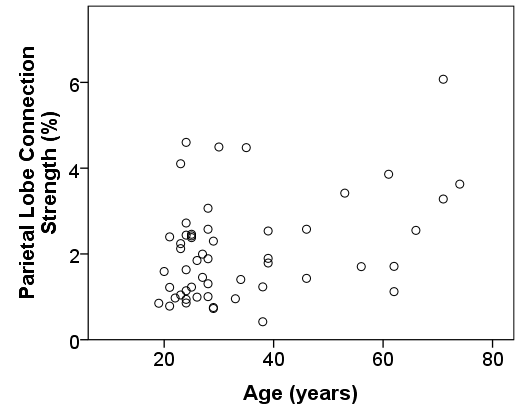


*Supplemental Figure 2. Significant findings in the Chronic Diseases Connectome Project cohort relating lobe-wise connection strength to age and gender.*

*Supplemental details - Detailed Hippocampal Anatomy*

Adjacent to the hippocampal formation in the inferior and lateral aspect is the parahippocampal gyrus (PHG), and anteriorly and superiorly (in the polar aspects) is the amygdala. Separating the amygdala and hippocampus head is the alveus which is an extension of the fimbria.

At a basic level, the hippocampus contains the Cornu Ammonis sections 1 to 4, the dentate gyrus, subiculum as well as the pre- and para- subiculum. Continuous with the hippocampus is the entorhinal cortex which is the anterior portion of the PHG. Within the hippocampus are various types of interconnecting fibers forming a complex and mostly uni-directional loop, including mossy fibers, performant pathways, and Schaffer collaterals. The entorhinal cortex (EC) is sometimes considered as part of the hippocampal formation due to its intricate connectivity with the hippocampus via collaterals and its well-established role as an input relay to the hippocampus, but for the present study it was not considered as part of the hippocampus. The EC is a major association area of archicortex, and as such has wide-reaching connections.

Other than the EC, a major WM connection between the hippocampus and other brain regions is via the alveus and fimbria (afferent myelinated fibers which “coat the hippocampus and contour its trajectory through the MTL”, ([Amaral, Park et al. 2016](#_ENREF_2))) and then (posteriorly) the fornix, which are continuations of one another. That it, the amygdala is separated from the head of the hippocampus by the alveus, which posteriorly converges on the medial surface to form a flattened band (the fimbria), which then forms the fornix at the level where the hippocampal body transitions into the tail. It then proceeds as an arch in the third ventricle that lies just below the corpus callosum (CC).

This WM band then communicates with subcortical structures such as the mammillary bodies (situated in the caudobasal part of the hypothalamus), nucleus accumbens, thalamus, and septal nuclei (sometimes called septal area; ([Nieuwenhuys, Voogd et al. 1988](#_ENREF_28)). Along with the nucleus accumbens, the septal nuclei are considered a pleasure zone in animals and are therefore often targets of deep brain stimulation for refractory psychiatric illness. In particular, the medial septal complex projects immensely to the hippocampus ([Swanson and Cowan 1979](#_ENREF_36)). The hippocampus (directly or indirectly) is also connected to the prefrontal/orbitofrontal cortices, cingulum, perirhinal cortices, striatum, amygdala, and hypothalamus, as well as raphe nuclei, periaqueductal gray, and the locus coeruleus ([Kawamura, Hattori et al. 1982](#_ENREF_19), [Nieuwenhuys 1985](#_ENREF_27)). Through biochemical exchange (primarily of dopamine, acetylcholine, serotonin and noradrenaline, ([Sil'kis 2009](#_ENREF_34), [Laplante, Mnie-Filali et al. 2013](#_ENREF_21))), the hippocampus is known to influence many behaviors including attention, novelty, reward, and mood, and hence implicated in psychiatric disorders.

Joining the two hippocampi is the hippocampus commissure. This is essentially a joining point for the two posterior crura (or crus) of the fornix, exquisitely demonstrated ex vivo by ([Pascalau, Popa Stănilă et al. 2018](#_ENREF_29)). Additionally, some fornical fibers traverse the anterior commissure (AC) to the contralateral hippocampus ([Schmahmann and Pandya 2009](#_ENREF_33)), and the tapetum (abutting the callosal bundle, although considered as part of the CC) provides connectivity to the homologous lateral and anterior temporal cortices ([Schmahmann and Pandya 2009](#_ENREF_33), [Mori, van Zijl et al. 2010](#_ENREF_26), [Pustina, Doucet et al. 2014](#_ENREF_30)). The AC, which crosses the midline in the lamina terminalis, is a critical interhemispheric pathway for other bilateral structures too, including the primary and secondary visual (V1 and V2) regions (e.g. ([van Meer, Houtman et al. 2016](#_ENREF_39))) as well as claustrum ([Arrigo, Mormina et al. 2017](#_ENREF_3)). The AC arches medially from the temporal lobes to reach the lamina terminalis and uses it as a bridge to cross the midline ([Smith 1972](#_ENREF_35)). From the lamina terminalis (column of the fornix) the fibres descend into the diencephalon. The AC has extensive connectivity to the occipital lobe as demonstrated in tractography.

The hippocampus is involved in many important tasks, including memory and learning ([Mack, Love et al. 2017](#_ENREF_23)) and spatial navigation ([Maguire, Woollett et al. 2006](#_ENREF_24)). As such, it has been directly linked to memory related disorders such as dementia ([Jaroudi, Garami et al. 2017](#_ENREF_18)) and psychiatric illnesses such as major depression and schizophrenia ([Maller, Daskalakis et al. 2012](#_ENREF_25)), as well as temporal lobe epilepsy ([Isnard and Bourdillon 2015](#_ENREF_17)) and mild traumatic brain injury ([Leh, Schroeder et al. 2016](#_ENREF_22)). It is unclear which part of the hippocampal formation is most effected in the acute and chronic stages of dementia and psychiatric illness, as the literature reports that all subfields may be implicated depending upon variables such as subgroup, years since diagnosis, age, and number of episodes (e.g. ([Cao, Passos et al. 2017](#_ENREF_9))).

*Detailed Hippocampal Connectivity*

The tractograms revealed connectivity to the AC via the fornix which enters the external capsule and comes into apposition with the inferior part of the claustrum ([Carpenter 1983](#_ENREF_10)), as seen in Figure 1C. As there were voxels containing both external capsule and the superior longitudinal fasciculus, the latter was also tracked. However, the external capsule is also connected with the SLF, hence it is possible that the SLF was tracked due to its connection with the external capsule.

Whilst the hippocampus is not directly connected to occipital regions, the hippocampus is connected to the entorhinal cortex (anterior PHG) via a network of synapses; this is why the hippocampus and EC are sometimes collectively referred to as the “hippocampal formation”. As the WM of the PHG is directly connected to the occipital lobe, the occipital lobe was revealed in the hippocampal tractography. Additionally, the AC is directly connected to the inferior longitudinal fasciculi; as the hippocampus tracked to the AC, it is likely that this is why the occipital fibres were tracked.

Alternatively, there are a number of possibilities that led to the optic radiations being tracked. Due to the tractography algorithms being bi-directional, WM connecting the occipital regions such as the primary visual cortex were also revealed, hence the generated pathways along the optic radiations extending anteriorly into the lateral geniculate nuclei. Furthermore, the AC connects to V1 and V2 ([van Meer, Houtman et al. 2016](#_ENREF_39)). Additionally, as the tapetum is between major optic radiation fibres in the region of the collateral trigones (sagittal stratum and corticofugal fibers, Figure 1C), visual pathways were also tracked. Finally, the tapetum in the region of the Meyer’s loop abuts the tapetum, and as the tapetum was tracked, Meyer’s loop was tracked which then propagated along the entire optic radiation ([Rasmussen 1943](#_ENREF_31)).

Medially, tractography propagated to the nucleus accumbens and thalamus, as well as the septal area. As the fornix curves medially, they enter the thalamus with most fibers terminating in the mammillary bodies (as described by ([Nieuwenhuys, Voogd et al. 1988](#_ENREF_28))).

WM fibres of the posterior limb of the internal capsule (PLIC) were tracked as well. This is likely due the PLIC being formed, in part, by fibres from thalamic nuclei ([Smith 1972](#_ENREF_35)). Furthermore, the posterior ventral thalamic nuclei connect to the spinothalamic tract ([Smith 1972](#_ENREF_35)). Additionally, the anterior ventral thalamic nucleus relays information to the area of the cerebral cortex that gives origin to the motor pathways ([Smith 1972](#_ENREF_35)). The pulvinar was also tracked, which contains fibres that too contribute to the makeup of the PLIC ([Smith 1972](#_ENREF_35)). A recent study which planted ROI seeds on the hippocampus and brainstem regions found that the tracks follow a latero-medial course and pass through the PLIC, then proceed through the ipsilateral cerebral peduncle, each bundle reaching midbrain, pons and bulb ([Arrigo, Mormina et al. 2017](#_ENREF_3)). That is, along the spinal-limbic pathway.

Tracks were revealed in the cerebellum traversing the Brachium Pontis, likely representing the dento-rubro-thalamic track and PLIC due to those fibres being connected to the hippocampus as described above. However, the connection may be more direct as the lamina terminalis descends into the diencephalon ([Smith 1972](#_ENREF_35)).

*Supplemental Table 1.*

*Published track density or super-resolution diffusion MRI studies.*

| Author | Subjects | MRI | Native resolution | Number of streamlines | Super-resolution | Results | Comments |
| --- | --- | --- | --- | --- | --- | --- | --- |
| CURRENT STUDY | N=1 | 3T, 1150d (multi-shell), CAIPI, b=500-2800 (11 shells), 32-channel, ASSET=2 | 2.0mm | 50 million | 300 micron | Highly detailed hippocampus connectivity and generation of hippocampus connectome | CSD with iFOD2, multi-shell |
| ([Aggarwal, Nauen et al. 2015](#_ENREF_1)) | Human cadaver brains (N=3) | 11.7T, 30d, b=2000 | 92 microns | Unknown | 10 microns | Able to visualize individual layers and bands of Baillarger | ~28 hrs scanning |
| ([Barajas, Hess et al. 2013](#_ENREF_4)) | 43 tumor specimens (ex vivo) | 3T, 55d, b=2000 | 1.8mm | 1 million | 250 microns | higher relative track density values of the tumor location yielded a higher likelihood of increased tumor proliferation, greater architectural disruption, and microvascular hyperplasia | Ex vivo tumor specimens |
| ([Calabrese, Badea et al. 2015](#_ENREF_5)) | Mice (N=2) | 9.4T, 120d, b=4000 | 43 micron | 5000 spv | 21 micron | Visualisation of tracts in mouse brain corresponding to neuronal tracer | 235 hrs scanning, 96 physical cores 1.5T RAM |
| ([Calamante, Tournier et al. 2010](#_ENREF_7)) | Human (N=5) | 3T, 20d, 60d, 150d, b=3000 | 2.3mm | 6 million | 125m (from 150d data) | First demonstration of enhanced signal with TDI |  |
| ([Calamante, Tournier et al. 2011](#_ENREF_6)) | Human (N=1), and phantom | 7T, 24-channel, ZOOPPA (net acceleration factor of 4.26), 60d, b=1000, NEX=6 | 1mm | 2 million | 800 micron | Showed that particular structures could be identified in the TDI maps only after using super  resolution | 7T, CSD with iFOD2 |
| ([Calamante, Tournier et al. 2012](#_ENREF_8)) | Mice (N=3) | 16.4T, 30d, b=5000 | 100 micron | 4 million | 20 micron | First to show the application of TDI to mouse brain imaging | 32 hrs scanning |
| Calamante (2012b) | Human (N=1) | 3T, 150d, b=3000 | 2.3mm | 60, 40, 6 million | 230 micron | Demonstrates novel image contrasts with TDI |  |
| Calamante (2013) | Humans (N=4) | 7T, 64d, b=2000, GRAPPA=3 | 1.8mm | 52 million | 200 micron | Showed markedly improved anatomical detail of the thalamic substructures | 7T |
| Calamante (2015) | Humans (N=8) | 3T, 60d, b=3000 | 2.5mm | 10 and 100 million | 2.5mm | Quantification of fiber density | Not SR because the study investigated reproducibility of TDI and effect of SIFT upon TDI. |
| Cho (2015) | Humans (N=4) | 7T, 64d, NEX=3, b=2000, GRAPPA=3 | 1.8mm | 6 million | 180 micron | Identified 4 fiber tracts that have not previously been directly visualized in vivo (septum pellucidum tract, anterior thalamic radiation, superolateral medial forebrain bundle, and inferomedial forebrain bundle) | 7T |
| Choi (2018) | Humans (N=3) | 7T, 64d, NEX=3, b=2000, GRAPPA=3 | 1.8mm | 6 million | 180 micron | Identified a newly observed anterior thalamocortical fiber of the thalamus | 7T |
| ([Dai, Wang et al. 2017](#_ENREF_11)) | Tree shrew (N=2) | 9.4T, 30d, b=5000 | 200 micron | 80 million (short tracks) and 8 million (long tracks) | 40 micron | Fine structures visible with TDI but not DTI | ~39 hrs scanning |
| ([Dhollander, Emsell et al. 2014](#_ENREF_12)) | Human (N=1) and phantom | 3T, 140d (multishell), b=700, 1000, 2800) | 2.5mm | 80 million | 200 micron | TDI (and track orientation density) is a versatile tool | Multi-shell |
| ([Farquharson, Tournier et al. 2016](#_ENREF_13)) | Human PVNH (N=14) + Controls (N=14) | 3T, 60d, b=3000 | 2.5mm | 1 million | 500 micron | TDI revealed abnormal fiber projections in nodular tissue suggestive of abnormal organization of WM |  |
| ([Garcia-Gomar, Soto-Abraham et al. 2017](#_ENREF_14)) | Human (N=12) | 3T, 120d, b=2000 | 2.00mm | 1 million | 200 micron | Anatomic characterization of prelemniscal radiations |  |
| ([Hamaide, De Groof et al. 2017](#_ENREF_15)) | Zebra finch (N=3) | 9.4T, 60d or 90d, b=2500 | 78 micron | 100 million | 40 micron | TDI in-line with histological investigations | ~44 hrs scanning |
| ([Hoch, Chung et al. 2016](#_ENREF_16)) | Human (N=3) | 3T, 256d, b=2500, acceleration=2, plus blipped (reverse phase-encoding) sequence | 3.00mm | 4 million | 500 micron | Revealed exquisite anatomic detail at 7 canonical levels of the brain stem | Improved anatomical detail by combining dwi data with T2 data |
| ([Kurniawan, Richards et al. 2014](#_ENREF_20)) | Mice (N=7+5) | 16.4T, 30d, b=5000 | 48 and 100 micron over 3mm iso block | 10 million tracks 10 times (equates to 100 million) | 20 and 10 micron | TDI enables 3-dimensional characterization of complex structures | 16.4T, ~32 hrs (N=7) and ~15 hrs with 1.5 zero-fill Fourier encoding acceleration (partial FT) in phase dimensions (N=5) |
| Palesi (2016) | Human (N=15) | 3T, 32-channel, 61d, b=1200 | 2.00mm | 2.5 million streamlines | 1mm | TDI enabled precise reconstruction of the cerebello-thalamo-cortical pathway | Not very high super-resolution |
| ([Richards, Calamante et al. 2014](#_ENREF_32)) | Mice (N=3) | 16.4T, 30d, b=5000 | 100 micron | 50 and 100 million | 20 micron | TDI connectivity maps were concordant with results obtained using anterograde dye tracing | 16.4T, ~32 hrs scanning |
| ([Toselli, Tortora et al. 2017](#_ENREF_37)) | Human (N=50) | 1.5T, 34d, b=1000 | 1.75mm x 1.75mm x 2.00mm | 20 million | 500 micron | TDI maps obtained presented higher anatomical detail than eigenvector maps | 1.5T; The aim of this work was to compare the results of DTI with those of TDI maps and CSD-PT on data from neonates and children, acquired with low angular resolution and low b-value diffusion sequences commonly used in pediatric clinical MRI  examinations |
| ([Ullmann, Calamante et al. 2015](#_ENREF_38)) | Zebrafish (N=4) | 16.4T, 30d, b=5000 | 48 micron | 60 million | 5 micron | Visualized 17 structures  were previously unidentifiable using MR microimaging | ~46 hrs scanning |
| Wenz (2016) | Human (N=18) | 3T, 64d, b=900, GRAPPA=2 | 2mm | 800,000 | 430 micron | Identification of brainstem nuclei |  |
| ([Willats, Raffelt et al. 2014](#_ENREF_40)) | Human (N=8) | 3T, 12-channel, 60d, b=3000 + b=0 blipped (reverse-phase encoded) | 2.5mm | 5 million | 625 micron | High within-subject reproducibility is an essential property for utility and sensitivity of TDI maps in clinical patient-control or longitudinal studies. |  |
| ([Wu, Reisinger et al. 2014](#_ENREF_41)) | Mice (N=7) + ex vivo (N=1) | 11.7T, 30d, b=2335, + (ex vivo) 30d, b=4000 | 100 micron | 1 million | 10 micron | TDI had similar findings to postmortem | ~20 hrs scanning |
| ([Ziegler, Rouillard et al. 2014](#_ENREF_42)) | PD (N=27) + Controls (26) | 3T head only, 120d, b=1000 and b=2500 | 2.4mm | 5 million | 1mm | Significant increases in TD were found | Previous version of MRtrix used (0.2.12) |

b=diffusion value (s/mm^2^), d=diffusion directions, CAIPI=Controlled Aliasing In Parallel Imaging, CSD=Constrained Spherical Deconvolution, iFOD2=2nd order integration over Fibre Orientation Distributions, GRAPPA=GeneRalized Autocalibrating Partial Parallel Acquisition, hrs=hours, PVNH=PeriVentricular Nodular Heterotopia, iso=isotropic, NEX=Number of EXcitations, spv=samples per voxel, SIFT=Spherical-deconvolution Informed Filtering of Tractograms, SR=Super-Resolution, T=Tesla, TD=Track Density.

**References**

Aggarwal, M., D. W. Nauen, J. C. Troncoso and S. Mori (2015). "Probing region-specific microstructure of human cortical areas using high angular and spatial resolution diffusion MRI." Neuroimage **105**: 198-207.

Amaral, R. S., M. T. Park, G. A. Devenyi, V. Lynn, J. Pipitone, J. Winterburn, S. Chavez, M. Schira, N. J. Lobaugh, A. N. Voineskos, J. C. Pruessner, M. M. Chakravarty and I. Alzheimer's Disease Neuroimaging (2016). "Manual segmentation of the fornix, fimbria, and alveus on high-resolution 3T MRI: Application via fully-automated mapping of the human memory circuit white and grey matter in healthy and pathological aging." Neuroimage.

Arrigo, A., E. Mormina, A. Calamuneri, M. Gaeta, S. Marino, D. Milardi, G. P. Anastasi and A. Quartarone (2017). "Amygdalar and hippocampal connections with brainstem and spinal cord: A diffusion MRI study in human brain." Neuroscience **343**: 346-354.

Barajas, R. F., Jr., C. P. Hess, J. J. Phillips, C. J. Von Morze, J. P. Yu, S. M. Chang, S. J. Nelson, M. W. McDermott, M. S. Berger and S. Cha (2013). "Super-resolution track density imaging of glioblastoma: histopathologic correlation." AJNR Am J Neuroradiol **34**(7): 1319-1325.

Calabrese, E., A. Badea, G. Cofer, Y. Qi and G. A. Johnson (2015). "A Diffusion MRI Tractography Connectome of the Mouse Brain and Comparison with Neuronal Tracer Data." Cereb Cortex **25**(11): 4628-4637.

Calamante, F., J. D. Tournier, R. M. Heidemann, A. Anwander, G. D. Jackson and A. Connelly (2011). "Track density imaging (TDI): validation of super resolution property." Neuroimage **56**(3): 1259-1266.

Calamante, F., J. D. Tournier, G. D. Jackson and A. Connelly (2010). "Track-density imaging (TDI): super-resolution white matter imaging using whole-brain track-density mapping." Neuroimage **53**(4): 1233-1243.

Calamante, F., J. D. Tournier, R. E. Smith and A. Connelly (2012). "A generalised framework for super-resolution track-weighted imaging." Neuroimage **59**(3): 2494-2503.

Cao, B., I. C. Passos, B. Mwangi, H. Amaral-Silva, J. Tannous, M. J. Wu, G. B. Zunta-Soares and J. C. Soares (2017). "Hippocampal subfield volumes in mood disorders." Mol Psychiatry **22**(9): 1352-1358.

Carpenter, M. B., Sutin, J. (1983). Human Neuroanatomy. Baltimore, MD, Williams & Wilkins.

Dai, J. K., S. X. Wang, D. Shan, H. C. Niu and H. Lei (2017). "Super-Resolution Track-Density Imaging Reveals Fine Anatomical Features in Tree Shrew Primary Visual Cortex and Hippocampus." Neurosci Bull.

Dhollander, T., L. Emsell, W. Van Hecke, F. Maes, S. Sunaert and P. Suetens (2014). "Track orientation density imaging (TODI) and track orientation distribution (TOD) based tractography." Neuroimage **94**: 312-336.

Farquharson, S., J. D. Tournier, F. Calamante, S. Mandelstam, R. Burgess, M. E. Schneider, S. F. Berkovic, I. E. Scheffer, G. D. Jackson and A. Connelly (2016). "Periventricular Nodular Heterotopia: Detection of Abnormal Microanatomic Fiber Structures with Whole-Brain Diffusion MR Imaging Tractography." Radiology **281**(3): 896-906.

Garcia-Gomar, M. G., J. Soto-Abraham, F. Velasco-Campos and L. Concha (2017). "Anatomic characterization of prelemniscal radiations by probabilistic tractography: implications in Parkinson's disease." Brain Struct Funct **222**(1): 71-81.

Hamaide, J., G. De Groof, G. Van Steenkiste, B. Jeurissen, J. Van Audekerke, M. Naeyaert, L. Van Ruijssevelt, C. Cornil, J. Sijbers, M. Verhoye and A. Van der Linden (2017). "Exploring sex differences in the adult zebra finch brain: In vivo diffusion tensor imaging and ex vivo super-resolution track density imaging." Neuroimage **146**: 789-803.

Hoch, M. J., S. Chung, N. Ben-Eliezer, M. T. Bruno, G. M. Fatterpekar and T. M. Shepherd (2016). "New Clinically Feasible 3T MRI Protocol to Discriminate Internal Brain Stem Anatomy." AJNR Am J Neuroradiol **37**(6): 1058-1065.

Isnard, J. and P. Bourdillon (2015). "Morphological imaging of the hippocampus in epilepsy." Rev Neurol (Paris) **171**(3): 298-306.

Jaroudi, W., J. Garami, S. Garrido, M. Hornberger, S. Keri and A. A. Moustafa (2017). "Factors underlying cognitive decline in old age and Alzheimer's disease: the role of the hippocampus." Rev Neurosci **28**(7): 705-714.

Kawamura, S., S. Hattori, S. Higo and T. Matsuyama (1982). "The cerebellar projections to the superior colliculus and pretectum in the cat: an autoradiographic and horseradish peroxidase study." Neuroscience **7**(7): 1673-1689.

Kurniawan, N. D., K. L. Richards, Z. Yang, D. She, J. F. Ullmann, R. X. Moldrich, S. Liu, J. U. Yaksic, G. Leanage, I. Kharatishvili, V. Wimmer, F. Calamante, G. J. Galloway, S. Petrou and D. C. Reutens (2014). "Visualization of mouse barrel cortex using ex-vivo track density imaging." Neuroimage **87**: 465-475.

Laplante, F., O. Mnie-Filali and R. M. Sullivan (2013). "A neuroanatomical and neurochemical study of the indusium griseum and anterior hippocampal continuation: comparison with dentate gyrus." J Chem Neuroanat **50-51**: 39-47.

Leh, S. E., C. Schroeder, J. K. Chen, M. M. Chakravarty, M. T. Park, B. Cheung, S. C. Huntgeburth, N. Gosselin, C. Hock, A. Ptito and M. Petrides (2016). "Microstructural Integrity of Hippocampal Subregions Is Impaired after Mild Traumatic Brain Injury." J Neurotrauma.

Mack, M. L., B. C. Love and A. R. Preston (2017). "Building concepts one episode at a time: The hippocampus and concept formation." Neurosci Lett.

Maguire, E. A., K. Woollett and H. J. Spiers (2006). "London taxi drivers and bus drivers: a structural MRI and neuropsychological analysis." Hippocampus **16**(12): 1091-1101.

Maller, J. J., Z. J. Daskalakis, R. H. Thomson, M. Daigle, M. S. Barr and P. B. Fitzgerald (2012). "Hippocampal volumetrics in treatment-resistant depression and schizophrenia: the devil's in de-tail." Hippocampus **22**(1): 9-16.

Mori, S., P. C. M. van Zijl and K. Oishi (2010). MRI Atlas of Human White Matter Amsterdam, Elsevier Science.

Nieuwenhuys, R. (1985). Chemoarchitecture of the brain. Berlin Heidelberg, NY Tokyo, Springer.

Nieuwenhuys, R., J. Voogd and C. van Huijzen (1988). The Human Central Nervous System: A Synopsis and Atlas. Berlin Heidelberg Springer-Verlag.

Pascalau, R., R. Popa Stănilă, S. Sfrângeu and B. Szabo (2018). "Anatomy of the Limbic White Matter Tracts as Revealed by Fiber Dissection and Tractography." World Neurosurgery.

Pustina, D., G. Doucet, C. Skidmore, M. Sperling and J. Tracy (2014). "Contralateral interictal spikes are related to tapetum damage in left temporal lobe epilepsy." Epilepsia **55**(9): 1406-1414.

Rasmussen, A. T. (1943). "The extent of recurrent geniculocalcarine fibers (loop of Archambault and Meyer) as demonstrated by gross brain dissection." Anat Record **85**: 277-284.

Richards, K., F. Calamante, J. D. Tournier, N. D. Kurniawan, F. Sadeghian, A. R. Retchford, G. D. Jones, C. A. Reid, D. C. Reutens, R. Ordidge, A. Connelly and S. Petrou (2014). "Mapping somatosensory connectivity in adult mice using diffusion MRI tractography and super-resolution track density imaging." Neuroimage **102 Pt 2**: 381-392.

Schmahmann, J. D. and D. Pandya (2009). Fiber Pathways of the Brain. USA, Oxford University Press.

Sil'kis, I. G. (2009). "Characteristics of the functioning of the hippocampal formation in waking and paradoxical sleep." Neurosci Behav Physiol **39**(6): 523-534.

Smith, C. G. (1972). Basic Neuroanatomy. Toronto, Canada, University of Toronto Press.

Swanson, L. W. and W. M. Cowan (1979). "The connections of the septal region in the rat." J Comp Neurol **186**(4): 621-655.

Toselli, B., D. Tortora, M. Severino, G. Arnulfo, A. Canessa, G. Morana, A. Rossi and M. M. Fato (2017). "Improvement in White Matter Tract Reconstruction with Constrained Spherical Deconvolution and Track Density Mapping in Low Angular Resolution Data: A Pediatric Study and Literature Review." Frontiers in Pediatrics **5**(182).

Ullmann, J. F., F. Calamante, S. P. Collin, D. C. Reutens and N. D. Kurniawan (2015). "Enhanced characterization of the zebrafish brain as revealed by super-resolution track-density imaging." Brain Struct Funct **220**(1): 457-468.

van Meer, N., A. C. Houtman, P. Van Schuerbeek, T. Vanderhasselt, C. Milleret and M. P. Ten Tusscher (2016). "Interhemispheric Connections between the Primary Visual Cortical Areas via the Anterior Commissure in Human Callosal Agenesis." Front Syst Neurosci **10**: 101.

Willats, L., D. Raffelt, R. E. Smith, J. D. Tournier, A. Connelly and F. Calamante (2014). "Quantification of track-weighted imaging (TWI): characterisation of within-subject reproducibility and between-subject variability." Neuroimage **87**: 18-31.

Wu, D., D. Reisinger, J. Xu, S. A. Fatemi, P. C. van Zijl, S. Mori and J. Zhang (2014). "Localized diffusion magnetic resonance micro-imaging of the live mouse brain." Neuroimage **91**: 12-20.

Ziegler, E., M. Rouillard, E. Andre, T. Coolen, J. Stender, E. Balteau, C. Phillips and G. Garraux (2014). "Mapping track density changes in nigrostriatal and extranigral pathways in Parkinson's disease." Neuroimage **99**: 498-508.
